# Spurious prospective effect of physical activity on problematic smartphone use: a simulated reanalysis and comment on Zhao et al. (2024)

**DOI:** 10.3389/fpsyg.2024.1485660

**Published:** 2024-11-01

**Authors:** Kimmo Sorjonen, Bo Melin

**Affiliations:** Department of Clinical Neuroscience, Karolinska Institutet (KI), Stockholm, Sweden

**Keywords:** cross-lagged panel models, physical activity, problematic smartphone use, simulated data, spurious prospective effects, triangulation

## Abstract

Based on findings from analyses with cross-lagged panel models, Zhao et al. concluded that physical activity can reduce problematic smartphone use (PSU) among adolescents. Here, we simulated data to resemble the data used by Zhao et al. We used triangulation and fitted complementary models to the simulated data and found contradicting decreasing, increasing, and null effects of initial physical activity on subsequent change in PSU. These divergent findings suggest that it is premature to assume a decreasing effect of physical activity on PSU and the conclusion by Zhao et al. in this regard can be challenged. It is important for researchers to be aware that correlations, including adjusted cross-lagged effects, do not prove causality in order not to overinterpret findings, something that appears to have happened to Zhao et al. We recommend researchers to triangulate by fitting complementary models to their data in order to evaluate if observed effects may be due to true causal effect or if they appear to be spurious.

Based on a negative effect of prior physical activity on subsequent problematic smartphone use (PSU) when adjusting for prior PSU in analyses with cross-lagged panel models (CLPM), Zhao et al. (2024) concluded that physical activity reduces PSU among adolescents. However, it is well-established that prospective effects in CLPM may be spurious due to correlations with residuals and regression to the mean (e.g. Lucas, 2023). The effect of prior physical activity on subsequent PSU when adjusting for prior PSU would correspond to a causal parameter only if prior physical activity and prior PSU were exogenous, i.e., independent of each other (Vowels, 2023). However, it is apparent in Zhao et al. that these two prior measures were not independent of each other. Moreover, if subsequent PSU would be affected by an unobserved confounder in addition to prior PSU and physical activity, the causal effect of physical activity on PSU would be unidentifiable (Vowels, 2022, 2023). In general terms, having access to and analyzing longitudinal data, in Zhao et al.'s case from three waves of measurement, is not sufficient for causal inference (Rohrer and Murayama, 2023).

If concluding that a negative adjusted effect of prior physical activity on subsequent PSU indicated a reducing effect, Zhao et al. should, for consistency, expect a positive effect of prior physical activity on prior PSU when adjusting for subsequent PSU, which would indicate that high prior physical activity had counteracted high prior PSU and allowed individuals to reach the same subsequent PSU as individuals with lower prior PSU but also with lower prior physical activity. A negative effect of prior physical activity on prior PSU when adjusting for subsequent PSU would, according to this logic, indicate a paradoxical increasing effect of physical activity on PSU. Moreover, if physical activity has, as concluded by Zhao et al., a reducing effect on subsequent PSU, we should expect a negative effect of prior physical activity on the subsequent PSU—prior PSU difference. The objective of the present study was to evaluate these predictions.

We simulated data to have the same crucial characteristics (i.e., sample size and correlations between variables) as in the data used by Zhao et al. We did not use the empirical dataset as it was not available to us. The simulation and analyses were conducted with R 4.4.0 statistical software (R Core Team, 2024) using the MASS package (Venables and Ripley, 2002). The analytic script, which also generates the simulated data, is available at the Open Science Framework at https://osf.io/ub8x3/.

In agreement with conclusions by Zhao et al. and a hypothesis of a true decreasing effect, prior physical activity had a negative effect on subsequent PSU when adjusting for prior PSU (Table 1, column 1). However, contrary to the prediction above, prior physical activity also had a negative effect on prior PSU when adjusting for subsequent PSU (Table 1, column 2). This negative effect indicates that low, not high, prior physical activity had counteracted high prior PSU and allowed individuals to reach the same subsequent level of PSU as individuals with lower prior PSU but with higher prior physical activity. Also contrary to the prediction above, prior physical activity did not have a negative effect on the subsequent—prior PSU difference (Table 1, column 3).

**Table 1:** Effect of prior physical activity (PA) on (a) subsequent PSU when adjusting for prior PSU (column 1); (b) prior PSU when adjusting for subsequent PSU (column 2); (c) subsequent—prior PSU difference (column 3).

**Time**	**Effect**		
	β**(PA**_T_**, PSU**_T+1_**.PSU**_T_**)**	β**(PA**_T_**, PSU**_T_**.PSU**_T+1_**)**	β**(PA**_T_**, PSU**_T+1_**-PSU**_T_**)**
T1 and T2	−0.043 [−0.084; −0.003]^*^	−0.110 [−0.150; −0.070]^**^	0.050 [0.001; 0.099]^*^
T2 and T3	−0.159 [−0.200; −0.118]^**^	−0.213 [−0.253; −0.173]^**^	0.040 [−0.008; 0.088]

β, standardized regression effect; PA, physical activity; PSU, problematic smartphone use.

The variables are given in the order predictor, outcome, covariate.

^*^p < 0.05.

^**^p < 0.001.

The present findings of divergent decreasing, increasing, and null effects of prior physical activity on subsequent change in PSU indicate that the finding by Zhao et al. presumably was spurious and that their conclusion of a true reducing effect can be challenged. It is important for researchers to be aware that correlations, including adjusted cross-lagged effects, do not prove causality in order not to overinterpret findings, something that appears to have happened to Zhao et al. As is common in psychological research (Vowels, 2022), there was a mismatch between Zhao et al.'s apparently causal hypothesis about an effect of physical activity on PSU and the available correlational data and conducted analyses. Consequently, it would probably have been preferable if Zhao et al. had described their findings in terms of prediction rather than in terms of causality.

## Data Availability

The datasets presented in this study can be found in online repositories. The names of the repository/repositories and accession number(s) can be found below: The analytic script, which also generates the simulated data, is available at the Open Science Framework at https://osf.io/ub8x3/.
